# Factors associated with delayed hospital presentation for patients with acute stroke in Makkah: A cross-sectional study

**DOI:** 10.1097/MD.0000000000030075

**Published:** 2022-08-26

**Authors:** Amal M. Alkhotani, Aseel Almasoudi, Jawaher Alzahrani, Emad Alkhotani, Mamdouh Kalkatawi, Alaa Alkhotani

**Affiliations:** a Department of Medicine, Umm Al-Qura University, Makkah, Saudi Arabia; b Department of Medicine, King Abdulla Medical City, Makkah, Saudi Arabia; c Department of Pediatric, Heraa Hospital, Makkah, Saudi Arabia; d Department of Radiology, King Abdulla Medical City, Makkah, Saudi Arabia; e Saudi Board of Neurology, Department of Medicine, Al-Noor Specialist Hospital, Makkah, Saudi Arabia; f Department of Pathology, Umm Al-Qura University, Makkah, Saudi Arabia.

**Keywords:** acute ischemic stroke, awareness, delayed presentation, factors, treatment

## Abstract

Despite the recent advancements in the treatment of acute ischemic stroke, the delayed patient arrival to emergency department or hospital serve as crucial factor for the selection of appropriate intervention program. This study was aimed to identify factors associated with late hospital arrival for patients with acute ischemic stroke in Makkah, Saudi Arabia. A prospective cross-sectional study was carried out at Al-Noor Specialist Hospital among 98 enrolled patients with the mean age of 60.4 ± 10.3 years over the period of March 2019 and June 2019. The data were collected through review of patient records and interview of patients and attendants. Fifty-four of these (55%) presented early (within 4.5 hours) and 44 (45%) presented late (after 4.5 hours). Factor associated with late arrival included low educational level (*P* = .01) and unemployment status (*P* = .033). The relationship between time of presentation and computed tomography findings showed statis,tically significant relationship between the former and early computed tomography findings (*P* = .017). A statistically significant relationship between time of presentation and knowledge of stroke was also observed (*P* = .013). Increased public awareness is important in order to minimize the time between stroke onset and emergency room presentation.

## 1. Introduction

Acute stroke is a common neurological disease, with an incidence depending on specific national context.^[1]^ In Saudi Arabia, a recent systemic review estimated that the total annual incidence of stroke is 29 per 100,000 people (95% confidence interval [CI]: 15–47),^[2]^ however, the rate of occurrence is lower than those of higher income countries. For example, in Australia, the annual stroke incidence is about 76 per 100,000, while in New Zealand, it is approximately 119 per 100,000.^[3]^ However, the stroke incidence in Saudi Arabia is variable according to region, with higher incidence reported in Aseer region (57.64 per 100,000 persons per year; 95% CI: 57.57–57.70),^[4]^ while lower incidences were reported in Al Madinah (13.89 per 100,000 persons).^[5]^

Management of acute ischemic stroke has advanced in recent years, yet success is largely dictated by the time it takes for a patient to seek intervention after onset. The earlier a patient receives emergency care, the better the prognosis. Thrombolytic therapy with recombinant tissue plasminogen activator (rt-PA, alteplase) is recommended for patients with acute ischemic stroke presenting within 4.5 hours from onset.^[6]^ Although rt-PA for acute ischemic stroke has been approved since 1996, the rate of its utilization remains low. In the United States, its utilization is estimated to be between 1.8% and 2.4%,^[7,8]^ whereas in Saudi Arabia, this figure is much higher.

Several studies have been conducted in the past few years related to the identification of factors associated with the delayed hospital presentation of patients with acute stroke. For instance; Froehler et al^[9]^ in their study emphasized on the importance of emergency transportation being a considerably important factor for providing effective treatment to the patients with stroke. The study further outlined that in most cases, healthcare providers fail to provide the required treatment such as; mechanical thrombectomy which is an endovascular procedure contributing in improving the functionality in patients with ischemic stroke. Since the treatment is cost effective and time dependent, lack of emergency transportation may result in producing several negative outcomes on patient’s health.^[9–11]^ Al Khathaami et al^[12]^ further outlined that certain treatment measures such as; Tissue plasminogen activator is effective when provided within 4.5 hours of onset acute ischemic stroke. However, most of the patients remain untreated due to late presentation to the hospital in Riyadh, Saudi Arabia. According to the study findings, factors behind the patient’s late arrival included; patients being alone at the time of onset of stroke, patients not transported to the hospital in ambulance, patients been unaware of the experienced disease, long-term distance between hospital and patient’s residence, patient’s lack of knowledge, difficulty faced by patients in accessing to the healthcare measures etc. Other considerable factors were outlined in the study of Ashraf et al,^[13]^ who focused on factors associated with the timely treatment of patients in the hospital for treatment. These factors included; patients presented in the hospital within 4 hours of stroke, <15 km distance from the hospital, patients reaching directly to the stroke department, high educational status of patients, along with those with the history of coronary artery disease, presence of hemiplegia etc.^[13]^

In other countries such as; China, Jiang et al^[14]^ highlighted that the rate of prehospital delay in patients were equal or greater than 2 hours, specifically among those who experienced stroke for the first time. Findings indicated that timely treatments were possible for patients due to the patient reaching hospital on time, patients directly reaching to the emergency department, participants with sufficient knowledge regarding initial symptoms of the stroke etc. Lachkhem et al^[15]^ in their systematic review of 31 studies identified different factors that significantly influenced the timely care of stroke onset treatments. These factors were then divided into 4 categories including factors that are central to patients, resource-based factors, lack of training, and lack of coordination. With regard to the factors that are central to patients, Arulprakash and Umaiorubahan^[16]^ outlined that patients residing in the rural area, not reaching to the emergency department, and those not perceiving their symptoms to be serious were the significant predictors in the patient’s delay for the treatment in India.

Like any other region, Saudi Arabia still lags behind in decreasing the ratio of morbidity and mortality due to the prevalence of acute stroke in patients. According to Al Khathaami et al,^[17]^ Saudi Arabia fails to practice knowledge of stroke, resulting in the failure of providing adequate healthcare support to patients. Several factors are attributed to the fiasco of reducing the negative outcomes of the disease. However, factors that are focused to patients are the most crucial of all. A recent study estimated that around 8.6% of all acute ischemic stroke patients presenting to tertiary care hospital in Saudi Arabia received rt-PA. The major reason for underutilization is late arrival to the emergency room, since alteplase needs to be administered as soon as possible. In the aforementioned study, only 29.8% presented in time for such intervention.^[18]^ The study from Saudi Arabia is more recent in comparison to USA studies and is carried out in tertiary care center. No study assessed the rate of thrombolysis in nationally in Saudi Arabia.

Hence, this study aims to identify factors associated with late hospital arrival for patients with acute ischemic stroke in the Makkah region. Findings are important and will actively contribute in developing effective measures to promote knowledge amongst the local population. The study is further effective in highlighting factors that are critical in developing effective measures to reduce the negative outcomes initiated through the delayed hospital presentation among patients.

## 2. Materials and Methods

### 2.1. Study design and population

This is a cross-sectional observational study carried out between March 2019 and June 2019, in the Al-Noor Specialist Hospital is one of the largest Ministry of Health Hospital’s in Makkah, serving a large population in the city and the surrounding area. The sample size for the study was calculated using “RaoSoft” calculator and samples were collected prospectively and consecutively using convenience sampling technique. The study was conducted in accordance with Strengthening the Reporting of Observational Studies in Epidemiology guidelines. All patients admitted to the medical ward with acute ischemic stroke diagnosed by neurologist and imaging were included in the study. Patients were excluded if they were pilgrims, if their neurological symptoms were secondary to other causes like infection or demyelination, if they were unable to provide data (e.g., aphasic patients with no family around), or if they had suffered a prior stroke. Following the study population, a total of 98 patients with the mean age of 60.4 ± 10.3 years were included, which were divided into 2 groups, based on their time of presentation to the hospital. After enrollment, the study objective was explained to all participants by researcher and informed consent for participation was obtained prior to data collection. The study was initiated after approval from Umm Al-Qura University’s ethics and research committee (approval number HAPC-02-K-012-2019-01-260).

### 2.2. Data collection

Data were collected by reviewing patients files, while specific considerations were provided to the information related to patients’ demographic features, risk factors, living situations, education background, employment status, time of onset to presentation, type of deficit, initial computed tomography (CT) findings, repeated imaging findings, knowledge of the deficit, and knowledge of stroke. The data were collected and recorded on an Excel sheet, and for each response the frequency and percentage were calculated.

### 2.3. Statistical analysis

Data analysis was performed using the software SPSS (Statistical Package for the Social Sciences) version 20. Categorical variables were described using their absolute frequencies and percentages. To compare the proportion of categorical data, a chi-square test and Fisher exact test were used when appropriate. The level of statistical significance was set at 5% (*P* < .05), with a highly significant difference deemed present if a *P* value was ≤ .001.

### 2.4. Ethical considerations

Ethical approval was obtained from Umm Al-Qura University’s ethics and research committee, and consent was acquired from all patients or their families.

## 3. Results

A total of 98 patients were observed in this study. Among them, 52 patients were males and 46 were females. From the overall study sample, 54 were presented early that is, within 4.5 hours of the onset of stroke, whereas 44 of them were presented late that is, after 4.5 hours of the stroke occurrence. Besides, from the overall sample, 79 of them were living with their families, while majority of them were illiterate (n = 38) and unemployed (n = 50). Table 1 shows patient demographic data in relation to their arrival time, with statistically nonsignificant findings. While statistically significant difference was observed between the time of presence and education level (*P* = .01) and employment (*P* = .033).

**Table 1:** Relationship between time of presentation and demographic data.

	Time of presentation		Test	
	Early, N = 54 (%)	Late, N = 44 (%)	*χ* ^2^	*P*
Gender				
Male	32 (55.2)	20 (42.5)	1.855	.173
Female	22 (44.8)	24 (57.5)		
Age (yr)				
<50	5 (9.3)	4 (9.1)	0.049	.824
50–60	16 (29.6)	15 (34.1)		
61–70	18 (33.3)	9 (20.5)		
>70	15 (27.8)	16 (36.4)		
Living situation				
Live alone	3 (5.6)	5 (11.4)	2.677	.102
With spouse	4 (7.4)	7 (15.9)		
With family	47 (87)	32 (72.7)		
Education level				
Illiterate	15 (27.8)	23 (52.3)	6.618	.01*
Primary school	15 (27.8)	8 (18.2)		
Secondary school	8 (14.8)	8 (18.2)		
Tertiary institution	5 (9.3)	3 (6.8)		
University	11 (20.4)	2 (4.5)		
Employment				
Employed	8 (14.8)	6 (13.6)	8.674	.033*
Self employed	5 (9.3)	3 (6.8)		
Retired	20 (37)	6 (13.6)		
Unemployed	21 (38.9)	29 (65.9)		

* *P* < .05 is statistically significant.

Table 2 shows the prevalence of risk factors among patients with respect to the time of presentation. Findings indicated a statistically nonsignificant relationship between time of presentation and presence of risk factors. Besides, diabetes and hypertension were prevalent in all patients (yet with a nonsignificant difference between both groups).

**Table 2:** Relationship between time of presentation and risk factors.

Risk factors	Time of presentation		Test	
	Early, N = 54 (%)	Late, N = 44 (%)	*χ* ^2^	*P*
DM	36 (66.7)	24 (54.5)	1.5	.221
HTN	32 (59.3)	33 (75)	2.69	.101
Hyperlipidemia	3 (5.6)	8 (18.2)	Fisher	.06
Smoking	11 (20.4)	13 (29.5)	1.104	.293
Prior TIA	8 (14.8)	7 (15.9)	0.022	.881
Atrial fibrillation	4 (7.4)	2 (4.5)	Fisher	.688
IHD	4 (7.4)	0 (0)	Fisher	.125
Other cardiac condition	3 (5.6)	0 (0)	Fisher	.25

DM = diabetes mellitus, HTN = hypertension, IHD = ischemic heart disease, TIA = transient ischemic attack.

*P < .05 is statistically significant.

Table 3 shows the relationship between time of presentation and CT findings. According to the observations conducted, a statistically significant relationship between the former and early CT findings (normal CT lesions were significantly present among those with early presentation (*P* = .017), while established lesions were significantly higher among those with late presentation) was observed. Besides, majority of participants (n = 47) had normal CT findings at the time of presentation.

**Table 3:** Relationship between time of presentation and CT finding.

CT findings	Time of presentation		Test	
	Early, N = 54 (%)	Late, N = 44 (%)	*χ* ^2^	*P*
Early CT finding				
Normal	31 (57.4)	16 (36.4)	8.243	.017*
Early changes	17 (31.5)	13 (29.5)		
Established stroke	6 (11.1)	15 (34.1)		

CT = computed tomography.

* *P* < .05 is statistically significant.

Table 4 demonstrates the relationship between the time of presentation and the deficit. Pure motor weakness was the most common neurological manifestation in both groups (n = 43). Besides, a statistically nonsignificant relationship between time of presentation and deficits presentation was observed. Other important findings related to the patients’ knowledge of deficit and time of presentation are provided in Table 5. Majority of patients including those of the late arrival group, recognized that their disease needed urgent intervention. Also, a statistically nonsignificant relationship between time of presentation and knowledge of deficit was observed.

**Table 4:** Relation between time of presentation and deficit.

Deficit	Time of presentation		Test	
	Early, N = 54 (%)	Late, N = 44 (%)	*χ* ^2^	*P*
Pure aphasia	1 (1.9)	0 (0)	Fisher	>.999
Pure motor weakness	23 (42.6)	20 (45.5)	0.081	.839
Dysarthria	3 (5.6)	2 (4.5)	Fisher	>.999
Ataxia	1 (1.9)	1 (2.3)	Fisher	>.999
Loss of consciousness	1 (1.7)	2 (5)	Fisher	.586
Aphasia and weakness	5 (9.3)	2 (4.5)	Fisher	.454
Aphasia, weakness, and sensory loss	5 (9.3)	3 (6.8)	Fisher	.727
Dysarthria and weakness	6 (11.1)	11 (25)	3.262	.709
Weakness and sensory loss	2 (3.7)	1 (2.3)	Fisher	>.999
Weakness dysarthria sensory	7 (13)	2 (4.5)	Fisher	.18
Sensory loss	1 (1.9)	1 (2.3)	Fisher	>.999
Dysarthria and ataxia	0 (0)	1 (2.3)	Fisher	.449

*P < .05 is statistically significant.

**Table 5:** Relationship between time of presentation and knowledge of deficit.

	Time of presentation		Test	
	Early, N = 54 (%)	Late, N = 44 (%)	*χ* ^2^	*P*
Transient and will recover	7 (13)	11 (25)	4.162	.125
Need urgent attention	40 (71.4)	24 (54.5)		
No intervention is available	7 (13)	9 (20.5)		

*P < .05 is statistically significant.

Table 6 shows the relationship between the arrival time and patients’ awareness that their symptoms suggested stroke. A statistically significant relationship between time of presentation and knowledge of stroke (*P* = .013). Patients who recognized that their symptoms were those of stroke were more likely to present early.

**Table 6:** Relationship between time of presentation and knowledge of symptoms as indicative of stroke.

Knowledge of stroke	Time of presentation		Test	
	Early, N = 58 (%)	Late, N = 40 (%)	*χ* ^2^	*P*
Yes	22 (40.7)	7 (16.3)	7.745	.013*
No	32 (59.3)	36 (83.7)		

* *P* < .05 is statistically significant.

## 4. Discussion

In this study, majority of the included patients arrived within the 4.5-hour window considered as early, although the difference is limited owing to our small sample size. The researcher found that the major factor associated with late arrival was educational level, with illiterate patients most likely to present late. Additionally, employment status played a major role in treatment seeking, with retired patients more likely to present early and unemployed patients more likely to present late. It is probable that the 2 factors—education and employment—are related to each other, as illiterate patients are likely to be unemployed.

Study findings also revealed that patients who are aware that they have had a stroke are more likely to present early, while those not cognizant that their symptoms are secondary to stroke presented late. There were no significant relationships between a patient’s age, gender, living situation, and risk factors to the arrival time. Although majority of patients, even those who arrived late, knew that they required immediate medical attention, no statistically significant relationship was observed. Patients who arrived late had a stroke diagnosis confirmed by imaging, while patients with earlier presentation tended to receive normal imaging. One interesting finding is that around 11.1% of the patients who arrived early showed an established stroke on imaging, which may indicate earlier unrecognized onset.

Several studies, conducted in different countries have outlined diverse factors associated to the late arrival time in the case of stroke. A study from the United States identified that patients who were aware that they had a stroke are likely to present early (odds ratio [OR] = 2.13, 95% CI, 1.01–4.46). Other predictors were calling 911 (OR = 3.40, 95% CI, 1.70–6.82), and the diagnosis of transient ischemic attack (OR = 3.57, 95% CI, 1.50–8) or lacunar stroke (OR = 2.78, 95% CI, 1.02–7.69).^[10]^ Another study from South Korea revealed different factors associated with early arrival, including the diagnosis of atrial fibrillation (OR = 1.505; 95% CI, 1.168–1.939), high initial National Institute of Health Stroke Scale scores (OR = 1.037; 95% CI, 1.023–1.051) indicating severity of stroke, onset during daytime (OR = 2.799; 95% CI, 2.173–3.605), and transport by an emergency medical service (OR = 2.127; 95% CI, 1.700–2.661).^[19]^ However, in the current study, no significant relationship between the time of presentation and the severity of stroke was observed, as identified by the presenting symptoms.

In Saudi Arabia, a study from Riyadh identified different factors associated with late arrival, including being alone at stroke onset (OR = 10.4, 95% CI, 1.3–82.0), not being transported in an ambulance (OR = 0.3, 95% CI, 0.1–0.5), not knowing that they were experiencing a stroke (OR = 4.2, 95% CI, 2.0–8.7), and residing outside the city of Riyadh (OR = 30.0, 95% CI, 9.0–99.9).^[12]^ These findings are similar to those presented in the current study as the identification of stroke symptoms and signs act as an important predictor of early arrival. Notably, in the current study, assessments regarding whether or not patients arrived through emergency services or via private transport were observed.

A separate study conducted in Riyadh to assess public awareness of stroke showed that 64% of responders were able to define stroke correctly. The findings included that 45.9% believe stroke and brain death have the same pathologic mechanism and outcome, and 18.4% of the responders identified ≥3 stroke symptoms. This indicates a major deficit in stroke awareness initiatives.^[19]^ Over the last few years, there have been many campaigns to increase awareness of stroke in Saudi Arabia, and especially to emphasize the critical influence of time between onset and intervention. However, there remain significant gaps in public knowledge. Thus, it is necessary to increase general awareness among the Saudi population about stroke presentations, as well as the importance of time. Arulprakash and Umaiorubahan^[16]^ pinpointed that awareness related to the symptoms and risk factors associated with the disease must be provided to the masses for the early detection and arrival of patients to the healthcare centers leading toward either morbidity or delay in treatments. In addition, for the required improvement of the community awareness, it is important to devise health promotion strategies where education of the local physicians related to the importance of early referrals to healthcare centers is important.

The current study has important limitations, beginning with its small sample size, and the fact that it is from a single center. Further, the researcher did not focus at whether patients arrived through emergency services or via their own transport, which can be a fruitful consideration for future research.

## 5. Conclusions

The current study focused on the factors associated to the delayed hospital presentation for patients with acute stroke. A significant number of included patients arrived late at hospital after the onset of stroke. Following the study findings, low education level, unemployment status, and lack of knowledge of stroke symptoms are risk factors for delayed hospital arrival. Thus, based on the findings, a call for increasing public awareness, especially targeting high risk patients that emphasizes how vital it is to avoid delays in hospital presentation is crucial. Based on the discussion, it is further important to make sure the availability of useful ambulance services, to expedite the presentation of patients in the healthcare centers resulting in the improved management of stroke in Makkah.

Future researchers are suggested to identify the influence of resource-based factors, and those associated to the lack of training and coordination on delayed hospital presentation of patients with acute stroke.

## Acknowledgments

The authors acknowledge all the associated personnel, who, in any reference contributed in the completion of this study.

## Author contributions

**Conceptualization:** Alaa Alkhotani, Mamdouh Kalkatawi.

**Data curation:** Amal M. Alkhotani.

**Formal analysis:** Amal M. Alkhotani.

**Funding acquisition:** Alaa Alkhotani.

**Investigation:** Amal M. Alkhotani.

**Supervision:** Aseel Almasoudi, Jawaher Alzahrani.

**Validation:** Aseel Almasoudi, Jawaher Alzahrani.

**Visualization:** Emad Alkhotani.
